# Geometric morphometric analysis of the brainstem and cerebellum in Chiari I malformation

**DOI:** 10.3389/fnana.2024.1434017

**Published:** 2024-08-07

**Authors:** Ishan R. Perera, Malek Zahed, Sydney Moriarty, Zachary Simmons, Maya Rodriguez, Courtney Botkin, Taylor Dickson, Bradley Kasper, Kendyl Fahmy, Jonathan A. Millard

**Affiliations:** ^1^Edward Via College of Osteopathic Medicine, Blacksburg, VA, United States; ^2^Department of Biomedical Sciences, Edward Via College of Osteopathic Medicine, Blacksburg, VA, United States

**Keywords:** Arnold-Chiari malformation, geometric morphometric analysis, neck pain, foramen magnum, posterior cranial fossa

## Abstract

**Background:**

Chiari I malformation (CMI) is characterized by inferior descent of the cerebellar tonsils through the foramen magnum and is associated with headache and neck pain. Many morphometric research efforts have aimed to describe CMI anatomy in the midsagittal plane using classical measurement techniques such as linear dimensions and angles. These methods are less frequently applied to parasagittal features and may fall short in quantifying more intricate anatomy with fewer distinct homologous landmarks.

**Methods:**

Landmark-based geometric morphometric techniques were used to asses CMI morphology in five anatomical planes of interest.

**Results:**

Significant shape differences between CMI and age/sex-matched controls were found in the midsagittal (Pseudo-*F* = 5.4841, *p* = 0.001) and axial planes through the rostral medulla (Pseudo-*F* = 7.6319, *p* = 0.001). In addition to tonsillar descent, CMI principal component 1 (PC1) scores in the midsagittal protocol were associated with marked anterior concavity of the brainstem and generalized verticality of the cerebellum with anterior rotation of its anterior lobe. In the axial medulla/cerebellum protocol, CMI PC1 scores were associated with greater anterior–posterior (A-P) dimension with loss of medial-lateral (M-L) dimension.

**Discussion:**

These results suggest that CMI is associated with greater curvature of the brainstem and spinal cord, which may perturb normal neural activities and disrupt cerebrospinal fluid movements. Previous reports on the A-P diameter of the posterior fossa in CMI have conflicted; our findings of greater A-P cerebellar dimensionality with concomitant loss of width alludes to the possibility that more caudal aspects of the posterior cranial fossa are more bowl-like (homogenous in axial dimensions) and less trough-like or elongated in the M-L direction.

## 1 Introduction

Chiari malformation I (CMI) is associated with many abnormalities involving the hindbrain and skull base (Alperin et al., 2014; Houston et al., 2018; Lawrence et al., 2018; Hiremath et al., 2020). Radiologically, CMI is characterized by greater than 5 mm caudal descent of the cerebellar tonsils below the McRae line (basion-opisthion line) with 50–75% of patients demonstrating syringomyelia (Steinbook, 2004; Heiss et al., 2012; Houston et al., 2018; Hiremath et al., 2020; Kular and Cascella, 2022; Papachristou et al., 2022). CMI is considered to be a primary congenital malformation, although some have described an acquired form (O’Shaughnessy et al., 2006; Heiss et al., 2012). Estimates predict CMI occurs in every 1 in 1,000 births with a slight female predominance (Kular and Cascella, 2022). Patients suffering from CMI most commonly exhibit pain localized to the occipital and posterior cervical areas often worsened by straining or Valsalva; less commonly, CMI patients may experience upper motor neuron signs and cranial nerve dysfunction (Steinbook, 2004).

Many investigations of CMI utilize traditional morphometric techniques, including linear, angular, and area measurements (Eppelheimer et al., 2018; Biswas et al., 2019). These techniques have been helpful in assessing more subtle differences in anatomical locales among CMI populations. Often these studies feature measurement protocols of osseous and soft tissue features in the midsagittal plane with the aim of identifying additional metrics which may inform clinical decision making, better align symptomatology, or provide predictive value of long-term outcomes (Houston et al., 2018). In contrast, there is a relative paucity of reports aimed to assess parasagittal CMI anatomy, although recent efforts have recognized the importance of accounting for the parasagittal situation of the cerebellar tonsils (Ebrahimzadeh et al., 2022). Other attempts to account for multidimensional variability have included volumetric evaluation of the posterior cranial fossa, with many results supporting the prevalent idea that occipital bone hypoplasia results in crowding of the posterior cranial fossa and subsequent disruption of the natural movements of cerebrospinal fluid (CSF) in the subarachnoid space (Dagtekin et al., 2011; Vurdem et al., 2012; Bagci et al., 2013).

In the last few decades, geometric morphometric (GM) techniques have been developed and regularly applied to problems in biology and adjacent fields that aim to describe variation in physical form (Mitteroecker and Schaefer, 2022). These methods – with utility in two or three dimensions – typically apply a set of homologous landmark points to individuals or specimens within a sample, followed by the implementation of generalized Procrustes analysis (Rohlf and Slice, 1990). Generalized Procrustes analysis serves to translate, scale, and rotate coordinate points to maximize superimposition and precedes downstream ordination techniques or group comparison. Centroid size is the typical scale variable for GM analyses. Many applications of these methods seek to connect allometric scaling to shape variation, although other applications explicitly seek to exclude the element of size in deference to shape-only analysis. Since less biological shape information is forfeited, these techniques often offer a more nuanced quantification of form than that of traditional biometric methods.

The primary aim of this research is to improve on the current morphometric descriptions of the CMI brain by using GM techniques to ascertain a more global understanding of shape differences in the CMI brainstem and cerebellum. To our knowledge, only 2 GM studies have been published evaluating Chiari anatomy. Ocakoglu et al. used nine discrete cerebellum-only landmarks to compare Chiari and controls (*n* = 40), and another by Ocakoglu et al. used 10 discrete brainstem-only landmarks (*n* = 50) (Ocakoglu et al., 2019, 2021). We hope to build on these reports by increasing sample sizes, using semilandmarks in greater density to increase resolution, and adding four axial landmarking protocols. A second primary aim is to address the relative dearth of research specifically designed to evaluate parasagittal characteristics of CMI. In addition to the expected differences in tonsillar position, a GM analysis will also reveal other locations of anatomical variability which may be appreciated in the midsagittal plane. We predict that the congestion of the neural components around the foramen magnum and other osseous constraints in the posterior cranial fossa results in a ripple effect of deformation within the brainstem and cerebellum that may be detectable and describable by applying landmark-based GM techniques to neural elements visualized in axial sections.

## 2 Materials and methods

### 2.1 Participants

Magnetic resonance imaging (MRI) studies of 94 Chiari-affected females and age/sex-matched controls were provided by the Chiari1000 database at the University of Akron. Each deidentified patient file was imported into 3DSlicer (v.5.6.2) and assessed for resolution quality.

### 2.2 Imaging planes

The midsagittal plane and four axial planes through the brainstem and cerebellum were chosen for analysis. Midsagittal images were selected based on midline landmarks such as the cerebral aqueduct, cerebellar nodulus, and obex. Scans with inadequate visualization of relevant anatomy in the midsagittal plane were excluded from the final midsagittal image dataset. Minor parasagittal variability has shown to have a negligible effect on midsagittal research studies, although this error may have clinical consequences (Morkos et al., 2022).

**Figure 1:**
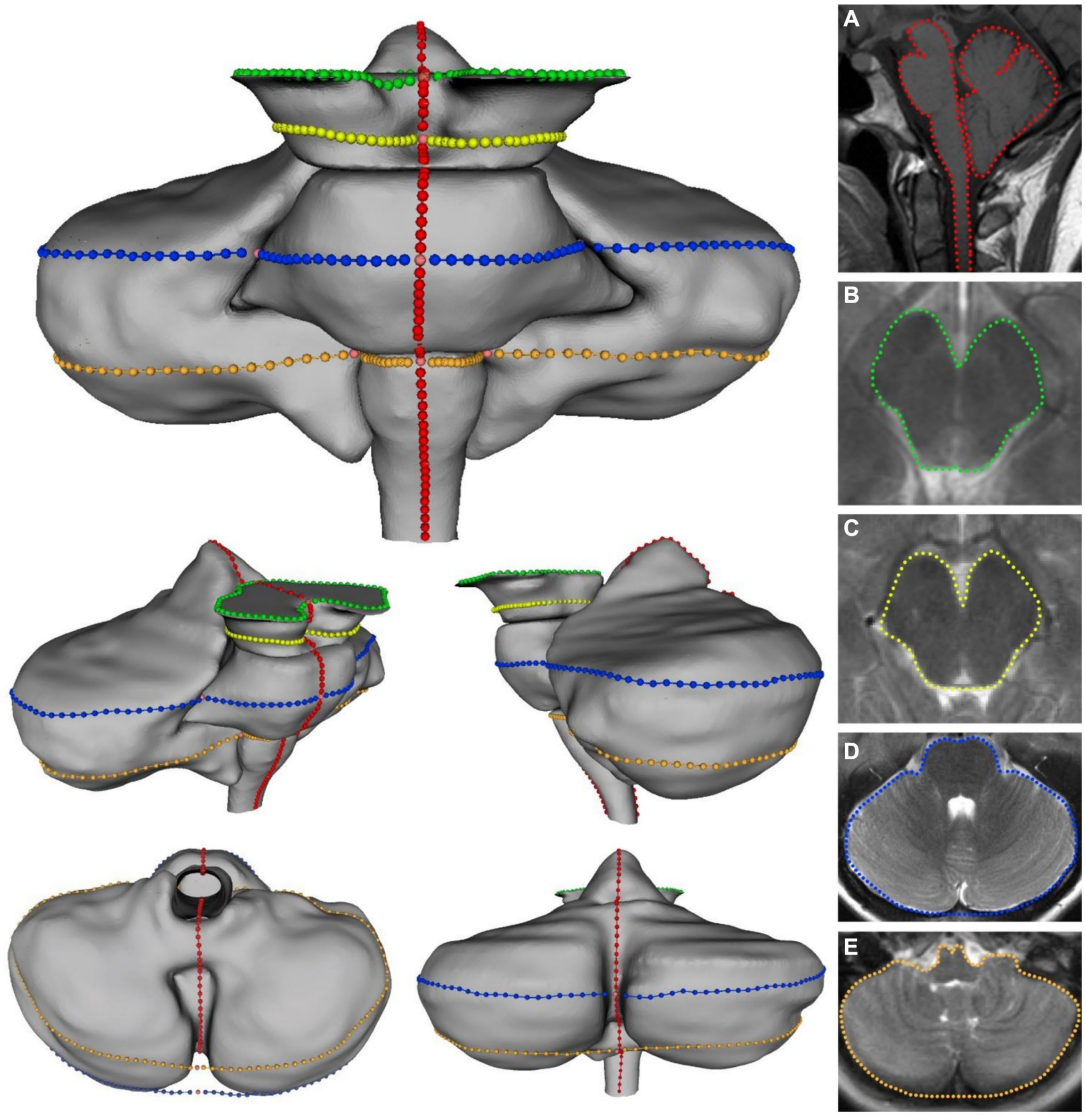
Visualization of the five landmarking protocols in 3D with corresponding 2D image for: **(A)** Midsagittal brainstem and cerebellum; **(B)** superior midbrain; **(C)** inferior midbrain; **(D)** pons/cerebellum; **(E)** medulla/cerebellum. Colors on 3D model correspond with colors in MRI images.

Prior to image selection for the axial plane landmarking protocols, the 3Dslicer *Transforms* module was used to translate and rotate each volume file to a modified Frankfort horizontal plane by aligning bilateral markers in the external acoustic meatus with bilateral markers placed at orbitale, ensuring homologous axial sectioning through each scan. Four axial imaging planes were selected through the brainstem and cerebellum. As tonsillar herniation and congestion around the foramen magnum are characteristic of CMI, these axial planes were elected to assess the potential for A-P and M-L shape deformation of the brainstem and cerebellum due to mass effect. Further, optimal planes of interest must offer representative coverage of the brainstem while simultaneously containing anatomical landmarks that can be reliably located between individual scans. The midbrain was assessed with two axial planes: one section of the superior midbrain through the level of the red nucleus and a second section of the inferior midbrain below the level of the red nucleus. These two selection criteria provided adequate coverage of the midbrain by producing image sets close to both ends of the midbrain. One axial plane was selected for the pons and mid-cerebellum. The relative homogeneity of pontine features on MRI made plane selection difficult, so a plane through the cerebellar nodulus was chosen to enhance reproducibility. In the transformed scans, this corresponded to sections approximating the mid-pons. Finally, a fourth axial section was selected through the superior medulla oblongata and inferior cerebellum. This plane was chosen as the most superior level of the medulla oblongata with distinct resolution, which frequently included the flocculus and features of the cerebellopontine angle. Images of poor quality or resolution were excluded from landmarking analysis. A description of the scans included in each protocol can be found in Table 1. Figure 1 illustrates the five planes selected for analysis.

**Table 1:** Description of sample.

Group	Sample (*n*)	Age at scan (*SD*)
Midsagittal		
Chiari	65	29.6 *(6.3)*
Control	29	32.0 *(4.0)*
Superior midbrain		
Chiari	54	29.9 *(6.5)*
Control	23	31.3 *(4.1)*
Inferior midbrain		
Chiari	65	30.1 *(6.4)*
Control	23	31.4 *(4.1)*
Pons/cerebellum		
Chiari	66	30.5 *(6.5)*
Control	24	31.5 *(4.1)*
Medulla/cerebellum		
Chiari	57	30.1 *(6.3)*
Control	24	31.5 *(4.1)*

Table describes mean and standard deviation values in parentheses for age at scan in years. Variable image quality between scans resulted in different sample sizes, as many images were excluded due to poor resolution.

### 2.3 Landmarking

Selected Chiari and control images were converted into TPS files using tpsUtil64 (v.1.82) and imported into tpsdig264 (v.2.32) for landmarking by blinded landmarkers. For each of the five datasets, anatomic landmarks were selected for reproducibility and fidelity, following descriptions by Bookstein (1991, pp. 55–87). Semilandmarks – which are usually a line of intermittent points or patches of points used to capture the shape of a curved line or surface – were also used to assess the outline shapes of the brainstem and cerebellum. As in our case, semilandmark curves or patches are typically positioned between landmarks with discrete anatomical loci (Gunz et al., 2005). Examples of discrete loci include single points that represent the intersection of tissues, the maxima of curved surfaces, or extremal locations. These point locations can be located accurately between scans.

Previously, semilandmark curves have been used to assess the complex morphology of the corpus callosum, which lacks many readily identifiable points of anatomical homology (Bookstein, 1997). A total of 165 landmarks and semilandmarks were placed on the midsagittal section, 82 were placed on both midbrain sections, and 134 were placed on both the pons/cerebellum and medulla/cerebellum sections. Landmarking protocols were designed to assess the outline shape of each level of interest, although the midsagittal landmarking scheme included independent outlining of the brainstem and cerebellum. Complete outlining of the brainstem and cerebellum allows for assessment of their relative positions in the posterior cranial fossa, which may be more informative when considering global differences in landmark configurations. Despite standardizing the plane of axial sectioning, sections of the pons and medulla demonstrated marked variability in the shape and space between cerebellar hemispheres. Additionally, the relative shape and location of the cerebellar vermis was highly variable between scans. This variation was controlled by treating the posterior aspects of the cerebellar hemispheres as a continuous curved surface, with no landmarks invaginating onto the interior contour of the cerebellar hemispheres. Functionally, this was necessary as there would not be reliable homologous anatomical locations between scans. It was difficult to take similar measures in the midsagittal landmarking scheme, so we anticipated that downstream ordination analyses may highlight this artifact of variation in the posterior lobe of the cerebellum. Each landmark was captured in the same order for each specimen to ensure homology between scans included in each sample. Depictions and descriptions of the landmarking protocol for each dataset can be found in Figure 1 and Table 2.

**Table 2:** Description of landmarks for all protocols.

Landmark no.	Definition
**Level of midsagittal plane**	
1	Ventral spinal cord at the axial intersection of the C2/C3 intervertebral disc
2	Ventral junction of the medulla and pons
3	Ventral junction of the pons and midbrain, within the interpeduncular fossa
4	Superior-most visible point of the ventral midbrain, within the interpeduncular fossa
5	Opening of cerebral aqueduct within the superior midbrain
6	Inferior cerebral aqueduct at the junction with the fourth ventricle
7	Dorsal spinal cord at the axial intersection of the C2/C3 intervertebral disc
8	Junction between cerebellar lobule I with the superior medullary velum
9	Deepest visible point within the primary cerebellar fissure
10	Junction between cerebellar lobule X and the superior medullary velum
11–30	20 semilandmarks between LM1 and LM2
31–45	15 semilandmarks between LM2 and LM3
46–50	5 semilandmarks between LM3 and LM4
51–55	5 semilandmarks between LM4 and LM5
56–60	5 semilandmarks between LM5 and LM6
61–90	30 semilandmarks between LM6 and LM7
91–115	25 semilandmarks between LM8 and LM9
116–165	50 semilandmarks between LM9 and LM10
**Level of superior midbrain**	
1	Ventral midsagittal point within interpeduncular fossa
2	Dorsal midsagittal point on tectum
3–42	40 semilandmarks between LM1 and LM2 on right
43–82	40 semilandmarks between LM1 and LM2 on left
**Level of inferior midbrain**	
1	Ventral midsagittal point within interpeduncular fossa
2	Dorsal midsagittal point on tectum
3–42	40 semilandmarks between LM1 and LM2 on right
43–82	40 semilandmarks between LM1 and LM2 on left
**Level of pons/cerebellum**	
1	Ventral midsagittal point within basilar groove
2	Midsagittal point approximating a continuous curve between cerebellar hemispheres
3	Junction between pons and cerebellum on the right
4	Junction between pons and cerebellum on the left
5–19	15 semilandmarks between LM1 and LM3
20–69	50 semilandmarks between LM3 and LM2
70–84	15 semilandmarks between LM1 and LM4
85–134	50 semilandmarks between LM4 and LM2
**Level of medulla/cerebellum**	
1	Ventral midsagittal point within anterior median fissure
2	Midsagittal point approximating a continuous curve between cerebellar hemispheres
3	Junction between medulla and cerebellum on the right
4	Junction between medulla and cerebellum on the left
5–19	15 semilandmarks between LM1 and LM3
20–69	50 semilandmarks between LM3 and LM2
70–84	15 semilandmarks between LM1 and LM4
85–134	50 semilandmarks between LM4 and LM2

LM, landmark.

### 2.4 Analysis

Raw landmark coordinates for each scan were imported into tpsRelw64 (v.1.5) where semilandmarks were allowed to slide along their respective curves to minimize the impact that arbitrary spacing between semilandmarks has on shape variation. Semilandmarks were slid to minimize the Procrustes distance between each scan, a least-squares method commonly practiced in GM techniques (Gunz and Mitteroecker, 2013). MorphoJ (v.1.07a) was used to generate a covariance matrix and perform a generalized Procrustes analysis for each sample. The symmetric component of shape was used for analyses of axial protocols. A principal component analysis (PCA) was used to explore the shape variation in each sample. All principal component (PC) scores for each sample were used to perform a permutational multivariate analysis of variance (PERMANOVA) to test for shape differences between Chiari and control groups (performed in R studio using the package *vegan*, function “adonis2,” version 2.6-4) (*α* = 0.05). PERMANOVA does not assume multivariate normality and is often more applicable in statistical shape analysis since the number of variables frequently outnumber the number of individuals in a sample (Anderson, 2001). PERMANOVA on all PC scores account for all related loadings between CMI and control groups for each protocol. A Mann–Whitney U test was performed to test for differences between groups for PC1 scores for each protocol. These results allow for direct comparison for higher impact PC axes, so group-specific shape interpretations may be made. Wireframe diagrams were created to visualize shape differences.

### 2.5 Error study

The reproducibility of each protocol was evaluated via protocol-specific error studies. One researcher landmarked the entire sample for each protocol. A second group of identical replicate scans were also landmarked: 25 replicates for the superior midbrain, inferior midbrain, pons/cerebellum, and medulla/cerebellum; the midsagittal protocol consisted of 11 replicates. Generalized Procrustes analysis was used for each sample and replicate group to determine the mean landmark configuration, and the total Procrustes distance was calculated for each scan. The mean Procrustes distance for each sample group was compared to that of the replicate group, with the expectation that a highly reproducible landmarking scheme would result in replicate groups having lower mean Procrustes distances with less dispersion (Robinson and Terhune, 2017).

## 3 Results

### 3.1 Principal component analyses

#### 3.1.1 Midsagittal

**Figure 2:**
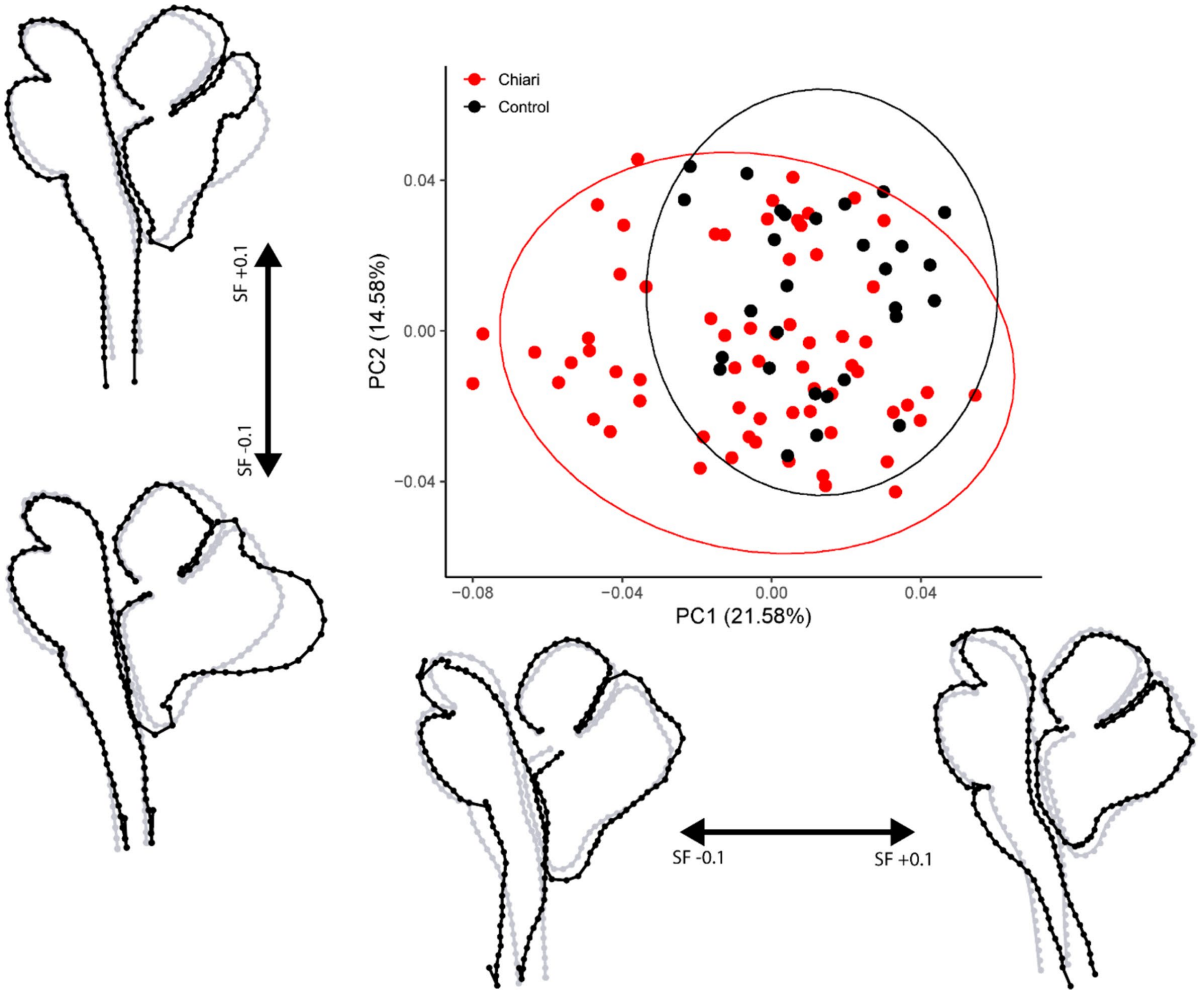
PCA plot with 95% confidence ellipses. Gray wireframes represent the mean landmark configuration, black wireframes represent shape changes along the respective PC axis. All scale factors are +/− 0.1. PC, principal component; SF, scale factor.

A total of 94 scans were included in this protocol (65 Chiari, 29 control). Principal component analysis for the midsagittal dataset revealed 93 principal components (PCs) with the first five eigenvalues accounting for 56.89% of the variance in the sample. Scores and shapes related to the first two PCs can be seen in Figure 2. The first principal component (PC1) captures 21.58% of the overall shape variation in the sample and is characterized by two notable shape features: first, concavity/convexity of the brainstem from the midbrain through the upper cervical spinal cord and second, the expected superior–inferior tonsillar movement. Shape conformations related to PC2 (14.58%) included modest anterior–posterior (A-P) flattening/bulging of the brainstem with remarkable A-P displacement of the posterior lobe of the cerebellum. In the cases of the first two PCs, more negative scores are associated with anterior displacement of the anterior lobe of the cerebellum, resulting in a more vertically oriented primary fissure.

#### 3.1.2 Superior midbrain

**Figure 3:**
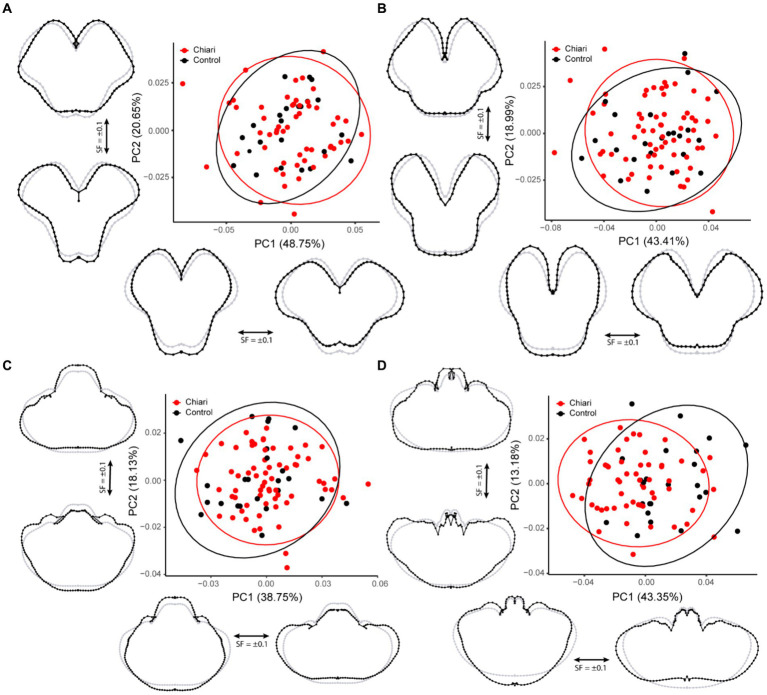
PCA plots with 95% confidence ellipses. Gray wireframes represent the mean landmark configuration, black wireframes represent shape changes along the respective PC axis. **(A)** Superior midbrain, **(B)** inferior midbrain, **(C)** pons/cerebellum, and **(D)** medulla/cerebellum. All scale factors are +/− 0.1. PC, principal component; SF, scale factor.

This protocol was implemented for 77 individuals (54 Chiari, 23 control). The first PC accounted for 48.75% of the variation in the sample, whereas PC2 accounted for 20.65% (Figure 3A). Shape changes along PC1 are characterized by widening/narrowing of the cerebral peduncles, leading to changes in the A-P dimensions of the midbrain. Shape changes highlighted by PC2 (20.65%) included medio-lateral (M-L) changes to the tegmentum resulting in a more (or less) pronounced tectum.

#### 3.1.3 Inferior midbrain

A total of 88 scans were included in this protocol, including 65 Chiari and 23 controls. The shape characterized by PC1 (43.41%) was similar that of the superior midbrain, widening/narrowing of the cerebral peduncles with changes in the A-P dimensions along the midline (Figure 3B). Shape conformations related to PC2 (18.99%) were also similar to the results from the superior midbrain protocol, including deep-set/protruding cerebral peduncles affecting overall tegmentum width and changes in the interpeduncular fossa.

#### 3.1.4 Pons/cerebellum

Ninety scans were included in this landmarking protocol (66 Chiari, 24 control). The first PC accounted for 38.75% of the variation in the shape within the sample and was notable for concomitant inverse changes in the A-P and M-L dimensions (Figure 3C). The second PC (18.13%) highlighted changes in the relative size of the pons, tissues around the cerebello-pontine angle, and more prominent changes to the petrous surface of the cerebellum.

#### 3.1.5 Medulla/cerebellum

A total of 81 scans were landmarked in this dataset (57 Chiari, 24 control). The first PC accounted for 43.35% of the overall shape variation and is notable for an inverse relationship between the A-P and M-L dimensions of the medulla/cerebellum (Figure 3D). Similar to the results from the pons/cerebellum protocol, PC2 (13.18%) showed that relatively larger medulla is associated with a cerebellum with a more prominent posterior M-L dimension, whereas a more recessed medulla is associated with a cerebellum with more prominent anterior M-L dimension.

### 3.2 Group comparisons

Results of the PERMANOVA revealed significant differences in PC scores between Chiari and control groups for the midsagittal (Pseudo-*F* = 5.4841, *p* = 0.001) and medulla/cerebellum (Pseudo-*F* = 7.6319, *p* = 0.001) configurations (Table 3). Shape configurations in more rostral axial sections were not significantly different between groups. Midsagittal Chiari PC1 (21.58%) scores clustered more on the negative end of the axis, which represented a higher degree of expected tonsillar displacement, along with marked anterior concavity of the brainstem and upper cervical spinal cord and greater anterior tilting of the anterior lobe of the cerebellum (*U* = 622, *p* = 0.003) (Figure 2). Medulla/cerebellum PC1 (43.35%) scores saw more negative Chiari scores relative to the control group (*U* = 328, *p* < 0.001) (Figure 3D). These negative scores were associated with reduced cerebellar width in favor of a greater A-P dimension which was most prominent at the cerebellopontine angle. More positive scores along the medulla/cerebellum PC1 axis were associated with greater cerebellar width and reduced A-P dimension. These results comport with our expectation that CNS shape deformation in the Chiari hindbrain may be detectable rostral to the typical area of interest around the foramen magnum.

**Table 3:** Results of PERMANOVA (number of permutations = 999).

Source	df	SS	MS	Pseudo-F	P(perm)
Midsagittal					
Group	1	0.02028	0.02028	5.4841	0.001*
Residuals	92	0.34025	0.00369837		
Total	93	0.36054			
Superior midbrain					
Group	1	0.002028	0.002028	1.4112	0.215
Residuals	75	0.107800	0.00143733		
Total	76	0.109829			
Inferior midbrain					
Group	1	0.003119	0.003119	1.8788	0.105
Residuals	86	0.142756	0.00165995		
Total	87	0.145875			
Pons/cerebellum					
Group	1	0.001051	0.001051	1.1587	0.307
Residuals	88	0.079793	0.00090674		
Total	89	0.080843			
Medulla/cerebellum					
Group	1	0.010099	0.010099	7.6319	0.001*
Residuals	79	0.104534	0.00132322		
Total	80	0.114632			

Significant results bolded and indicated by (*).

### 3.3 Error study

**Figure 4:**
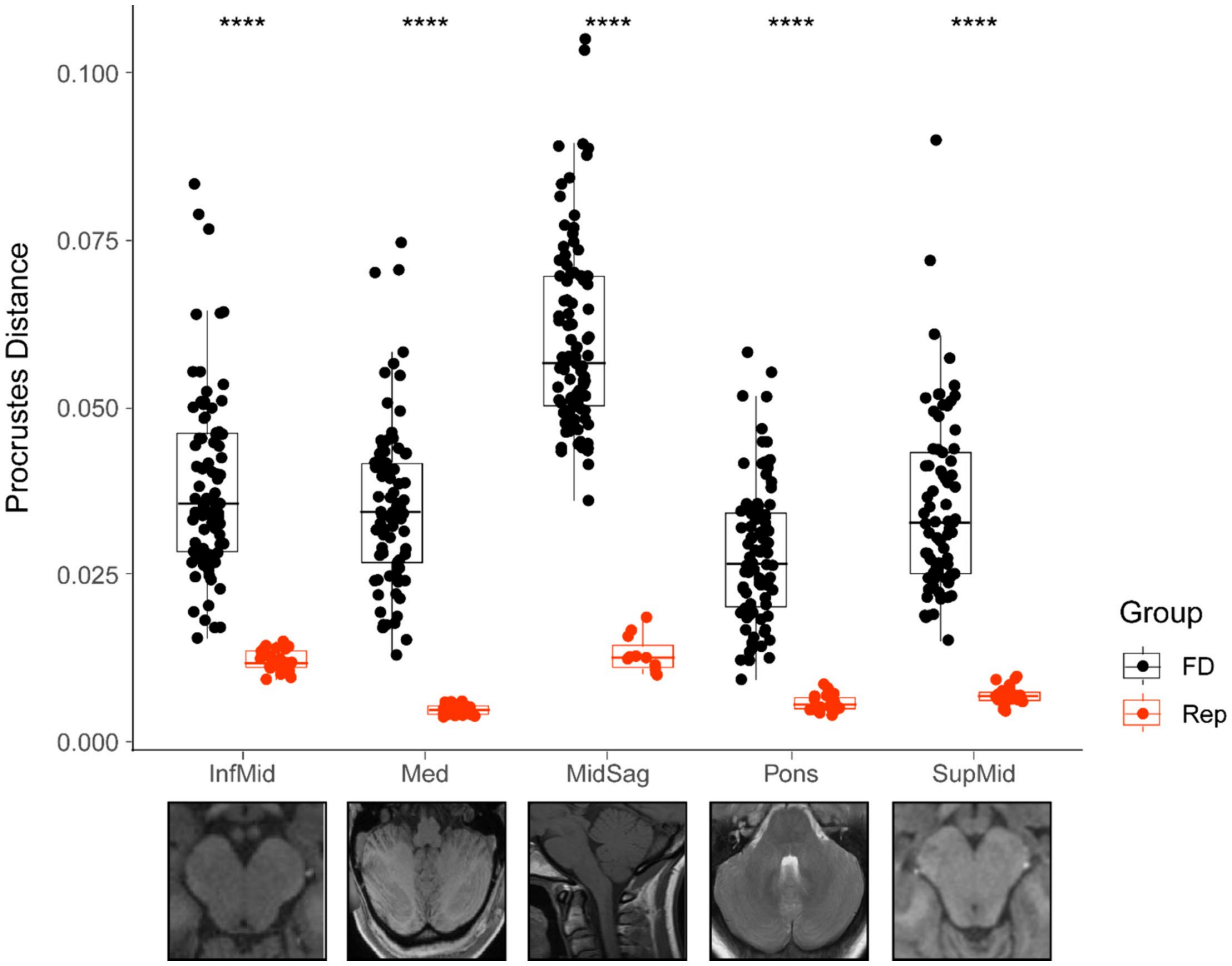
Comparison of Procrustes distances for each total sample and its corresponding replicate. Bars represent median value for each group, boxes show the interquartile range (25th to 75th percentile), and the extension of the whiskers represents 1.5 times the interquartile range. All group comparisons are significant (*p* < 0.001 represented by ****). FD, full dataset; Rep, replicate dataset.

Comparisons of the average Procrustes distance for each landmarked scan between the complete sample and its corresponding replicate set were significant for all groups (*p* < 0.001) (Figure 4). There was also much lower variation in each sample’s replicate group than in the complete sample. The midsagittal group had a higher average Procrustes distance (0.060 ± 0.014). Simply having more landmarks or greater natural variability in structures in the midsagittal plane may have accounted for this finding. Nevertheless, the midsagittal replicate average was relatively low (0.013 ± 0.002).

## 4 Discussion

Beginning with early morphometric descriptions of the posterior cranial fossa, many efforts have focused on revealing linear and angular differences in Chiari populations (Nishikawa et al., 1997; Milhorat et al., 1999; Urbizu et al., 2014). In this study, our approach is to enhance the current understanding of the shape of the Chiari brainstem and cerebellum by applying landmark-based GM techniques. Beyond exploring landmark configuration differences using PCA, we also sought to test for shape differences between Chiari and age/sex matched controls. The results of the PERMANOVA revealed significant differences between Chiari and control groups in the midsagittal plane and in axial section through the rostral medulla.

### 4.1 Review of midsagittal plane findings

In the midsagittal plane, the more negative CMI scores along PC1 (21.58%) correspond with greater degrees of tonsillar ectopia, which was expected. Perhaps the most notable midsagittal shape feature for more negative PC1 scores was the marked anterior concavity of the entire brainstem and upper cervical spinal cord. Greater odontoid retroversion and retroflexion have been demonstrated in females and individuals with CMI, and odontoid invasion of the vertebral canal is significantly larger in CMI populations (Besachio et al., 2015; Houston et al., 2018). Additionally, the Boogard angle, the angle between McRae’s line and the clivus, is larger in individuals with CMI (Houston et al., 2018). These reports are congruent with our quantitative description of the brainstem and upper cervical spinal cord’s anterior curvature, as the tissue must allow for bending to naturally comply with cranial nerve penetration into the dura mater or similar fixed locations like the internal acoustic meatus or porus trigeminus. It should be noted that the interface of the lower cranial nerves with the skeleton of the posterior fossa may not be consistent between individuals. For example, variation in shape of the internal acoustic meatus and porus trigeminus have been demonstrated (Ogut et al., 2021; Sekerci et al., 2021). These local changes may also have a relationship with brainstem orientation.

Since the curvature seen in our results is accompanied by tonsillar descent, a close approximation of the cerebellar vermis with the brainstem was also found. These combinations of shape features seen in the CMI midsagittal plane may contribute to disruption of CSF flow between the fourth ventricle and surrounding subarachnoid space. While most interest in CMI CSF flow disruptions are focused on the foramen magnum, it should be worth considering that perturbations caused by the shape conformations we have described may prohibit natural CSF movement through foramen Magendie, foramina Luschka, or through the central canal of the spinal cord (Heiss, 2023). Investigation into the relationship between shape conformation and the incidence of syringomyelia would further reveal the role of hindbrain shape in CMI CSF flow.

Research aimed at elucidating the pathophysiology of CMI may often be confounded by the presence of basilar invagination, which is characterized by superior displacement of the odontoid and resultant compression or crowding around the foramen magnum. Various reports aimed at clinical management of these potentially coincident conditions have been published (Goel et al., 1998; Klekamp, 2015). In a more recent morphometric study, Wang et al. isolated CMI patients from basilar invagination patients and still found an increased clival angle coupled with decreased clivoaxial angle in CMI patients compared to control (Wang et al., 2020). These findings were similar to the Boogard angle (identical to “clival angle”) and Wackenheim angle (similar to “clivoaxial angle”) reported by Houston et al. (2018). This skull base flattening with concurrent anterior inclination of the brainstem may be seen in CMI-only individuals without any other skeletal abnormalities around the craniocervical junction.

Aside from tonsillar changes, more negative midsagittal PC1 (21.58%) scores were also associated with generalized verticality of the cerebellum. Increased midsagittal cerebellar height has been previously reported in CMI patients (Biswas et al., 2019). Our results add greater resolution to the global midsagittal accommodations of the cerebellum, showing that in CMI the posterior lobe tends to be far less deeply situated in the posterior cranial fossa, while the anterior lobe is forced anteriorly with the primary fissure becoming less horizontally oriented. Shape changes associated with midsagittal PC2 (14.58%) scores localize heavily on the posterior cerebellum. We predicted the sensitivity of our methods would likely detect natural variability of the curvature of the posterior cerebellum, internal occipital crest, and meningeal dura mater. Compared to axial landmarking protocols, it was much more difficult to account for this variation in a rigorous fashion that did not add unnecessary distortion to the results, so instead we opted to anticipate this artifact in the results.

### 4.2 Review of axial plane findings

The other significant result showed shape differences between Chiari and control groups in the axial plane through the superior medulla. Chiari configurations clustered along the more negative medulla PC1 (43.35%) axis, which was suggestive of an increase in the A-P cerebellar dimension with concomitant loss of cerebellar width. More positive medulla PC1 scores demonstrated a much wider cerebellum, with less A-P cerebellar dimension, notably around the cerebellopontine angle. This axis of shape variation represented a significant amount of variability in the overall sample (43.35%). There are conflicting reports about CMI differences in posterior cranial fossa A-P diameter, with most linear measurements taken at or close to internal occipital protuberance to the superior limit of the clivus (Aydin et al., 2005; Houston et al., 2018).

Our results suggest a more circular-shaped medulla/cerebellum in CMI as opposed to an oval medulla/cerebellum. It is likely that the ultimate deformation of the CMI brainstem/cerebellum at this level is due to a multitude of influences, including congestion at foramen magnum and other osseous constraints related to hypoplasia of the occipital bone. It is important to consider that the shape in A-P and M-L dimensions illustrated by the results of the axial landmarking occurs concurrently with the shape in A-P and superior–inferior dimensions depicted by the midsagittal landmarking protocol. The shape of the posterior cranial fossa may play an important role in pathology of CMI, regardless of posterior fossa volume or metrics of scale. It is possible that the more oval or “trough-like” medulla/cerebellum shape associated with the control group is a consequence of a similarly shaped posterior fossa, which may offer more M-L cerebellar support. As the CMI group was associated with a more circular medulla/cerebellum at that level, a similarly shaped posterior fossa may act more like a funnel, offering less support and functioning more like a drain. Future morphological studies may specifically address these possibilities by targeting skeletal components.

The lack of significant differences between CMI and control groups in more superior landmarking configurations suggests that any gross compression or deformation forces on the natural outline boundary captured in the axial plane are diminished or negligible rostral to the pontomedullary junction.

### 4.3 Review of error study findings

Procrustes distances between each specimen and the mean configuration for each sample should be lower in a sample of replicates compared to a sample of different individuals. In this case, the Procrustes distance would simply be a measure of error, and therefore a useful proxy for the reliability of the landmarking strategy. These results suggest the landmarking protocol was robust and yielded accurate measurements, engendering confidence in the results.

## 5 Conclusion

In summary, this research has revealed subtle features of the midsagittal CMI brainstem and cerebellum which may be difficult to ascertain without the aid of landmark-based GM techniques. Some of these features corroborated previous evidence, while other features were newly described. Additional morphometric interest in parasagittal CMI anatomy is an opportunity for future morphometric applications, both traditional and geometric.

## 6 Limitations

There are many limitations to this study which are important to acknowledge. Although the strength of the methods lies in the amount of shape information retained by GM techniques, they were nevertheless employed in two dimensions. The outcomes of these landmarking strategies are only strictly applicable to the planes they are used to evaluate. One must be cautious when extrapolating beyond these planes. Despite the landmarkers being blinded to the identities of each scan and the results of the error studies being encouraging, there is still an element of landmarking error introduced into the analysis, which invariably influences the results. Lastly, our aims were not to evaluate skeletal components, so speculations about the precise nature of the skeletal shape influencing the shape of the brainstem and cerebellum must be drawn with caution.

## Data availability statement

The datasets presented in this study can be found in online repositories. Datasets are available on Figshare online repository. Coordinates for all five datasets after sliding of semilandmarks – https://doi.org/10.6084/m9.figshare.25843861 and PC scores for all five datasets https://doi.org/10.6084/m9.figshare.25843885.

## Ethics statement

This study involving humans was evaluated and approved by the institutional review board at the Edward Via College of Osteopathic Medicine (Project #1944161-1; VCOM IRB record #2022-049). The study was conducted in accordance with the local legislation and institutional requirements. Written informed consent for participation was not required from the participants or the participants’ legal guardians/next of kin in accordance with the national legislation and institutional requirements.

## Author contributions

IP: Data curation, Investigation, Writing – original draft. MZ: Data curation, Investigation, Writing – review & editing. SM: Data curation, Investigation, Writing – review & editing. ZS: Data curation, Investigation, Writing – review & editing. MR: Data curation, Investigation, Writing – review & editing. CB: Data curation, Investigation, Writing – review & editing. TD: Data curation, Investigation, Writing – review & editing. BK: Data curation, Investigation, Writing – review & editing. KF: Data curation, Investigation, Writing – review & editing. JM: Conceptualization, Formal analysis, Funding acquisition, Methodology, Project administration, Resources, Supervision, Visualization, Writing – review & editing.
